# Incidence, Causes, and Timing of Unplanned Reoperations Following Three‐Column Osteotomy for Pediatric and Adult Spinal Deformities: A Long‐Term Single Center Study

**DOI:** 10.1111/os.70190

**Published:** 2025-11-05

**Authors:** Youping Tao, Chenhao Zhao, Jigong Wu, Jiaxu Wang, Bo Gao, Haixia Li, Yongyu Hao, Litao Huo, Shibo Huang, Zhiming Chen, Shuwei Ma, Shuilin Shao

**Affiliations:** ^1^ Department of Spine Surgery, the Ninth Medical Center of PLA General Hospital Beijing China; ^2^ The Second Affiliated Hospital of Anhui Medical University Hefei Anhui Province China

**Keywords:** complications, pedicle subtraction osteotomy, scoliosis, three‐column osteotomy, unplanned reoperation, vertebral column resection

## Abstract

**Objective:**

Unplanned reoperation is regarded as one of the most critical indicators for evaluating healthcare quality. To date, long‐term data sets on unplanned reoperations following three‐column osteotomy (3CO) for spinal deformity patients remain largely limited. To uniquely investigate the incidence, causes, and timing of unplanned reoperations following 3CO procedures for pediatric and adult spinal deformities, with a focus on long‐term follow‐up outcomes.

**Methods:**

We retrospectively analyzed data of all consecutive pediatric and adult spinal deformities who underwent single‐posterior 3CO procedures between 2013 and 2016 from a single scoliosis center. The timing of revisions was categorized as perioperative (0–6 weeks), short‐term (6 weeks–2 years), intermediate‐term (2–5 years), and long‐term (> 5 years). Statistical analyses were performed using the Mann–Whitney *U* test for continuous variables and chi‐squared test or Fisher's exact test for categorical variables between patients with and without unplanned revision surgery.

**Results:**

Among the 216 patients who underwent 3CO procedures, with a median follow‐up duration of 10 years (interquartile range, 9–11), 36 patients (15 pediatric and 21 adult cases) required unplanned revision surgeries, yielding an overall reoperation rate of 16.7%. The top three causes of revision were implant failure (10 cases, 4.6%), deformity progression (nine cases, 4.2%), and pseudarthrosis (8 cases, 3.7%). Other reasons included new neurological deficits (four cases, 1.9%), infection (two cases, 0.93%), wound dehiscence (two cases, 0.93%), and acute coronal imbalance (1 case, 0.46%). Of the 36 patients who underwent revisions, 22.2% underwent revision during the perioperative period, 13.9% in the short term, 33.3% in the intermediate term, and 30.6% in the long term. Patients undergoing multilevel 3CO exhibited significantly higher unplanned revision rates than those undergoing single‐level 3CO (*p* < 0.05).

**Conclusion:**

This study found that the overall unplanned reoperation rate following 3CO for spinal deformities was 16.7%. Implant failure, deformity progression, and pseudarthrosis were the most common reasons. Multilevel 3CO was significantly associated with a higher likelihood of unplanned revision surgery.

**Trial Registration:** Chinese Clinical Trial Center (registration number: ChiCTR2500096201, available online at www.chictr.org.cn/).

This study provides one of the most extensive long‐term follow‐up analyses of unplanned revisions following 3CO for spinal deformities, with a mean follow‐up duration of 9.8 years, which has been underexplored in prior research. The findings provide critical data for preoperative discussions with patients and their families regarding the risks of unplanned reoperation. Additionally, the study highlights the need for long‐term surveillance and proactive strategies to mitigate revision risks, particularly in patients undergoing multilevel 3CO.

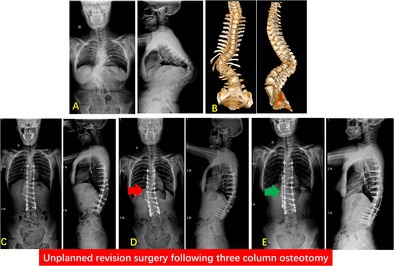

## 1 Introduction

Spinal deformity, such as scoliosis and kyphosis, can significantly affect the quality of life in both pediatric and adult patients. Three‐column osteotomies (3CO) are advanced surgical techniques used to correct complex spinal deformities. These procedures mainly include pedicle subtraction osteotomy (PSO), Scoliosis Research Society‐Schwab Grade IV osteotomy (SRS‐Schwab 4), hemivertebrectomy, and vertebral column resection (VCR) [1, 2, 3, 4, 5, 6, 7, 8, 9, 10, 11, 12]. Notably, 3CO has been increasingly employed for spinal deformities with diverse etiologies such as congenital deformity, post‐tuberculous kyphosis, idiopathic scoliosis, and ankylosing spondylitis [12, 13, 14, 15, 16, 17, 18]. However, despite their effectiveness, 3CO procedures are associated with high complication rates, such as implant failure, deformity progression, pseudarthrosis [19, 20, 21, 22, 23, 24, 25, 26, 27, 28, 29, 30, 31], and even permanent paraplegia in severe cases [18, 20, 21]. Many of these complications necessitate unplanned revisions, which further complicate patient outcomes [4, 5, 6, 11, 12, 13, 16, 24, 31, 32, 33].

Unplanned reoperations impose considerable socioeconomic burdens on patients, families, and healthcare systems [34, 35, 36, 37, 38, 39, 40], and their incidence is now a key metric for evaluating healthcare quality [38]. Reflecting this priority, China's National Health Commission explicitly mandated reducing unplanned reoperation rates in its 2025 National Medical Quality and Safety Improvement Targets (Target IX: NIT‐2025‐IX) (March 2025). While prior studies report reoperation rates following 3CO—e.g., 12.3% at 3 months and 17.6% at 1 year (adults) [13], 19% within 1 year (adults) [35], 37.5% over 4.6 years (pediatric congenital) [4], and 28% at ≥ 5 years (mixed cohorts) [5]—they primarily focus on short‐ to intermediate‐term outcomes (30 days–5 years) or involve limited sample sizes. Consequently, long‐term data on unplanned reoperations—including incidence, etiology, and temporal patterns—remain largely unexplored in contemporary spine literature.

An in‐depth understanding of the unplanned revisions following 3CO therapy is crucial for clinical practice. For instance, such data can serve as a valuable reference for surgeons, enabling more informed preoperative discussions with patients regarding the risks of unplanned reoperation and facilitating the development of strategies to minimize revision surgery [22]. Therefore, the purposes of this study are (i) to uniquely investigate the incidence, causes, and timing of unplanned reoperations following 3CO for pediatric and adult spinal deformities with a focus on long‐term follow‐up outcomes; and (ii) to compare demographic and surgical parameters between patients with and without unplanned revision surgery and to examine potential risk factors for unplanned revision.

## 2 Materials and Methods

### 2.1 Study Design and Ethical Approval

This single‐center retrospective study was approved by our institutional ethics committee (ethical approval number: LL‐LCSY‐2024‐14). Owing to the retrospective nature of the study, the requirement for formal consent was waived.

### 2.2 Patients Information

We retrospectively reviewed the electronic medical records of 246 consecutive pediatric and adult patients (aged 18 years and older) with spinal deformity who underwent single‐stage posterior 3CO procedures with pedicle screw‐only constructs between January 2013 and December 2016 at a single spinal deformity center. Exclusion criteria comprised patients with spinal intradural tumors, post‐traumatic spinal deformities, or degenerative deformities. The exclusion of degenerative deformities aimed to minimize confounding from age‐related comorbidities.

### 2.3 Selection of Surgical Methods and Indications

In this study, all patients underwent single‐stage posterior 3CO using a pedicle screw‐only construct. The specific 3CO techniques performed included: PSO, VCR, SRS‐Schwab Grade 4 Osteotomy, and hemivertebrectomy [1, 2, 3, 4, 5, 6, 7, 8, 9, 10, 11, 12]. The choice of technique was individualized based on the type and severity of deformity, vertebral level, surgeon's expertise, and patient‐specific anatomical considerations. All pedicle screws were inserted using a freehand technique based on previous investigations [7, 41, 42]. In addition, all 3CO surgeries were performed by the same senior surgical team.

### 2.4 Data Collection

The following data were extracted and recorded: diagnosis of spinal deformity, age at the time of surgery (years), sex, height (cm), weight (kg), body mass index (BMI, kg/m^2^), history of prior spinal deformity surgery, number of levels fused, number of 3CO levels (single‐level or multilevel), operation time (minutes), estimated blood loss (mL), type of 3CO procedures, preoperative major coronal and sagittal Cobb angle, length of follow‐up period, and reasons for unplanned revision surgery. Unplanned revisions were defined as any subsequent surgical intervention related to the initial 3CO procedure that was not planned at the time of index surgery.

The interval between index surgery and reoperation was recorded. Based on the classification by Riley et al. [6], the timing of unplanned revision surgery was categorized into four periods: perioperative period (0–6 weeks), short‐term (6 weeks–2 years), intermediate‐term (2–5 years), and long‐term (> 5 years). For patients with multiple reasons for reoperation, the primary cause was recorded and used in subsequent data analyses [43].

To identify potential risk factors for unplanned revision surgery, demographic and surgical parameters (e.g., age at primary surgery, operation time, and number of levels fused) were compared between patients with and without unplanned revision surgery.

### 2.5 Statistical Analysis

In this study, continuous variables such as age, BMI, and operation time are shown as median with interquartile range (IQR). Group comparisons for these parameters were conducted using the Mann–Whitney *U* test. Categorical variables were analyzed using the chi‐squared test or Fisher's exact test, as appropriate. Statistical significance was defined as *p* < 0.05. All analyses were performed using GraphPad Prism 9.5.1 (San Diego, CA, USA) and IBM SPSS Statistics version 27.0 (IBM Corp., Armonk, NY, USA).

## 3 Results

### 3.1 Demographic Data

A total of 216 spinal deformity patients who underwent the 3CO procedure via a single‐stage, posterior‐only approach with pedicle screw‐only constructs were included in this analysis, comprising 84 pediatric patients and 132 adult patients. The cohort consisted of 131 females (60.6%) and 85 males (39.4%), with a median age of 21 years (IQR, 13–28). Index surgery was the primary procedure in 192 patients (88.9%) and revision in 24 patients (11.1%). Among the patients, 192 (88.9%) underwent single‐level 3CO and 24 (11.1%) underwent multilevel 3CO.

For the entire cohort (*n* = 216), the median operation time and estimated blood loss was 422 min (IQR, 328–491) and 2050 mL (IQR, 1000–3000), respectively. The median number of spinal fusion levels was 12 (IQR, 7–13). The median preoperative major Cobb angles in the coronal were 80° (IQR, 50‐110) and 80° (IQR, 50–108) in the sagittal plane. All patients were followed up for a minimum of 8 years, with a median follow‐up duration of 10 years (IQR, 9–11).

Regarding the diagnosis of spinal deformities, 132 (61.1%) patients had congenital spinal deformity, 33 (15.3%) had syringomyelia‐associated scoliosis, 29 (13.4%) had idiopathic scoliosis, 9 (4.2%) had ankylosing spondylitis, 7 (3.2%) had post‐tubercular spinal deformity, 3 (1.4%) had neuromuscular scoliosis, and 3 (1.4%) had spinal deformities associated with neurofibromatosis Type 1.

The 3CO techniques performed included SRS‐Schwab Grade 4 osteotomy in eigh cases (3.7%), PSO in 97 cases (44.9%), VCR in 60 cases (27.8%), and hemivertebral resection in 51 cases (23.6%). Detailed demographic data are presented in Table 1.

**TABLE 1:** Comparison of patient characteristics and surgical data between the patients with and without unplanned revision surgery.

Variables	Revision (*n* = 36)	No revision (*n* = 180)	Total (*n* = 216)	*p*
Age (years)	23 (7–36)	21 (13–27)	21 (13–28)	0.82
Sex (male/female)	16/20	69/111	85/131	0.49
Height (cm)	143 (130–157)	147 (138–153)	147 (137–154)	0.54
Weight (kg)	47 (21–56)	44 (37–52)	44 (35–53)	0.95
BMI (kg/m^2^)	23 (17–25)	20 (18–23)	20 (18–23)	0.20
Etiology				
Congenital	24	107	131	0.56
Idiopathic	5	24	29	
Syringomyelia‐associated	2	30	32	
Ankylosing spondylitis	2	7	9	
Post‐tubercular	2	5	7	
Neuromuscular	1	2	3	
Neurofibromatosis Type 1	0	3	3	
Preoperative main coronal curve angle	64 (39–94)	83 (51–110)	80 (50–110)	0.10
Preoperative main sagittal curve angle	83 (46–128)	80 (53–107)	80 (50–108)	0.49
Number of fusion levels	10 (4–12)	12 (7–13)	12 (7–13)	0.06
Operative time (minutes)	388 (264–488)	428 (341–492)	422 (328–491)	0.40
Estimated blood loss (ml)	1800 (625–3000)	2200 (1000–3000)	2050 (1000–3000)	0.31
History of prior spinal fusion surgery				0.77
No	33	159	192	
Yes	3	21	24	
Number of 3CO level				**0.02**
Single level	28	164	192	
Multilevel	8	16	24	
Types of 3CO procedure				0.13
SRS‐Schwab Grade 4	1	7	8	
PSO	10	87	97	
VCR	13	47	60	
Hemivertebrae resection	12	39	51	

*Note*: Continuous variables are shown as median with IQR in parentheses. *p* < 0.05 indicates statistical significance. Bold indicates statistically significant *p* values.

### 3.2 Unplanned Reoperation Rate and Causes

Among 216 patients who underwent 3CO procedures, 36 (16.7%) required unplanned revision surgery during the follow‐up period. This cohort comprised 15 pediatric cases (17.9% of 84 pediatric patients) and 21 adult cases (15.9% of 132 adult patients).

The top three reasons for unplanned revision surgery were implant failure in 10 patients (10/216 = 4.6%; 10/36 = 27.8%), deformity progression in nine patients (9/216 = 4.2%; 9/36 = 25.0%), pseudarthrosis in eight patients (8/216 = 3.7%; 8/36 = 22.2%), followed by new neurologic deficits in four cases (4/216 = 1.9%; 4/36 = 11.1%), infection in two cases (2/216 = 0.9%; 2/36 = 5.6%), wound dehiscence in two cases (2/216 = 0.9%; 2/36 = 5.6%), and acute coronal imbalance in one case (1/216 = 0.5%; 1/36 = 2.8%).

Among the 15 pediatric cases, the most common reasons for revision were deformity progression (seven cases) and implant failure (three cases), followed by new neurologic deficits (two cases), pseudarthrosis (two cases), and wound dehiscence (one case).

Among the 21 adult patients, the most common reasons were implant failure (seven cases) and pseudarthrosis (six cases), followed by infection (two cases), deformity progression (two cases), new neurologic deficit (two cases), wound dehiscence (one case), and acute coronal imbalance (one case).

**FIGURE 1:**
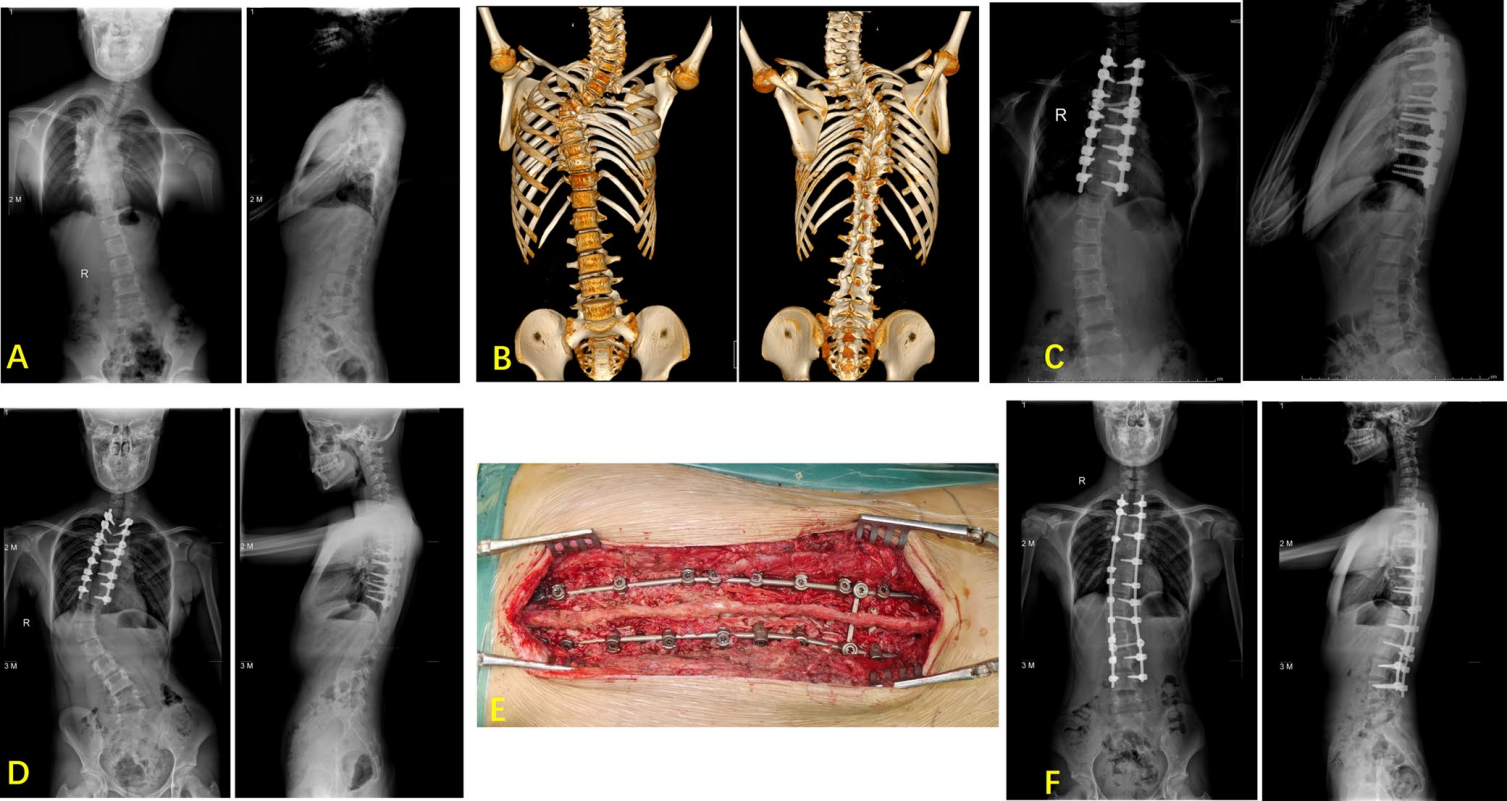
A 10‐year‐old female with congenital scoliosis. (A) Preoperative long‐standing scoliosis radiographs. (B) Preoperative three‐dimensional computed tomography scans showed congenital hemivertebra at T5 and T6. (C) Single‐stage posterior hemivertebra resection of T5 and T6, and bilateral pedicle screw fixation from T2 to T11 was performed. (D) Spinal deformity progression was found during the follow‐up. (E) Intraoperative photograph showed an unplanned revision surgery that was performed during intermediate term (2.4 years postoperatively). (F) The full‐spine radiographs after unplanned revision, with a distal fusion level to L4.

**FIGURE 2:**
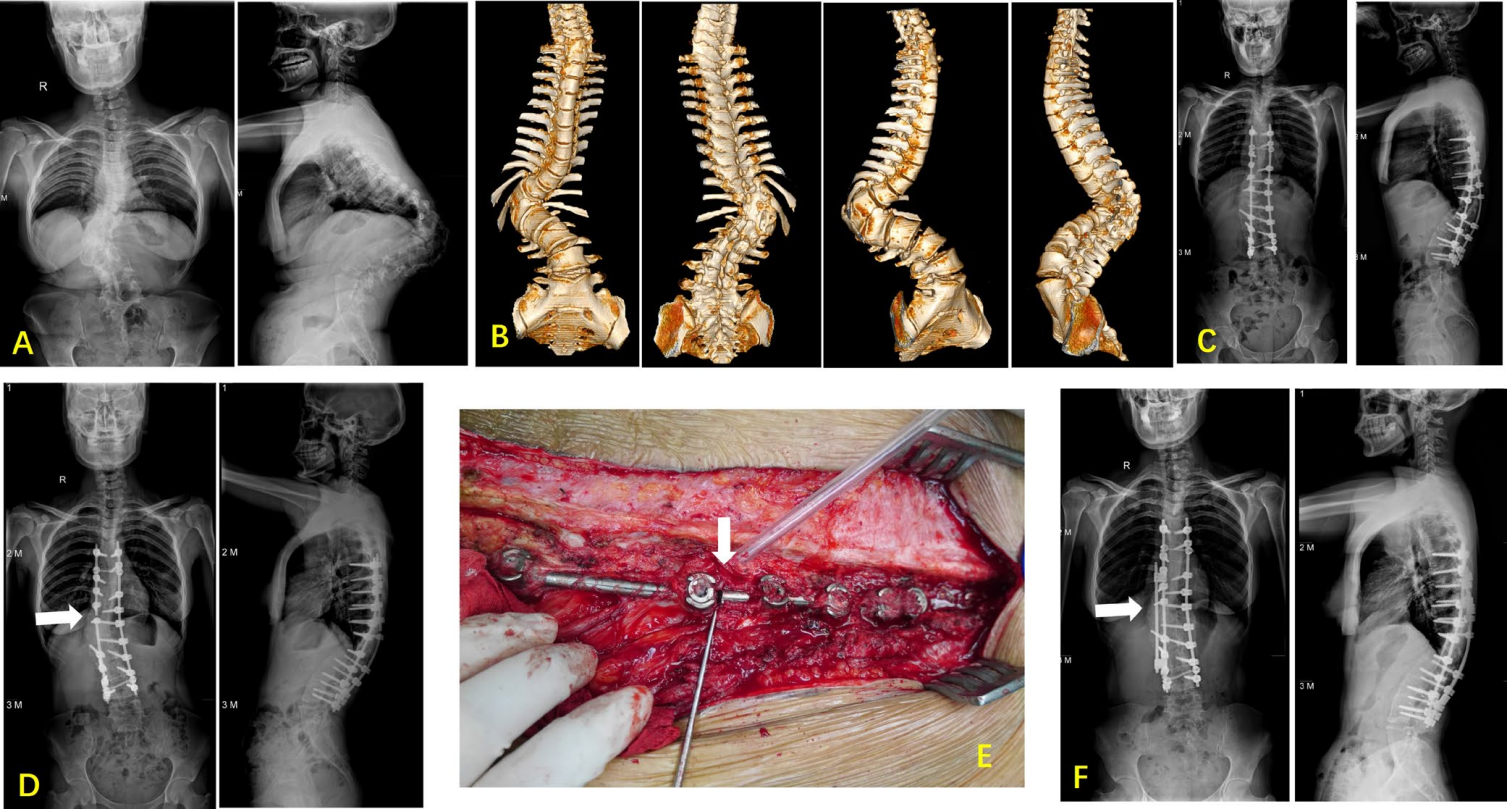
The patient was a 26‐year‐old female with congenital kyphoscoliosis. (A) Preoperative long‐standing scoliosis radiographs. (B) Preoperative three‐dimensional computed tomography scans. (C) Three‐column osteotomy and correction were performed, and good correction in coronal and sagittal plane deformity was obtained. (D) and (E) Postoperative and intraoperative photographs showed pseudarthrosis and implant failure during short term (20 months postoperatively), and an unplanned revision surgery was performed. (F) The full‐spine radiographs after unplanned revision, and the additional rod across the three‐column osteotomy were used.

In addition, among the 10 patients requiring reoperation due to implant failure, the specific causes included rod/screws breakage (five cases), painful instrumentation (two cases), screw displacement (one case), the rod separated from the pedicle screw due to the failure at the connector side (one case), and pedicle screw loosening (one case). Among the nine patients with deformity progression, seven patients (77.8%) were pediatric spinal deformity patients, with the majority occurring in congenital spinal deformity cases (8/9, 88.9%). (An example of unplanned reoperation due to deformity progression is seen in Figure 1). Additionally, the majority of pseudarthrosis cases (6/8, 75%) occurred in adults (an example of this is shown in Figure 2).

### 3.3 Timing of Unplanned Revision Surgery

The timing of unplanned revision surgeries following 3CO procedures was categorized as follows: (1) perioperative period (0–6 weeks), 8 patients (8/216 = 3.7%; 8/36 = 22.2%); (2) short‐term (6 weeks–2 years), 5 patients (5/216 = 2.3%; 5/36 = 13.9%); (3) intermediate‐term (2–5 years), 12 patients (12/216 = 5.6%; 12/36 = 33.3%); and (4) long‐term (> 5 years), 11 patients (11/216 = 5.1%; 11/36 = 30.6%).

Overall, 23 patients (23/216 = 10.6%; 23/36 = 63.9%) required unplanned reoperation beyond 2 years postoperatively.

#### 3.3.1 Perioperative Term (0–6 Weeks)

Among the eight patients requiring revision during this period, the most common reason was the new neurological deficit (four cases), including three cases requiring immediate reoperation on the same day after 3CO and one case requiring revision surgery on postoperative Day 10. Other reasons included wound dehiscence (two cases), infection (one case), and acute coronal imbalance (one case). No revisions were made for pseudarthrosis, deformity progression, or implant failure during this period. Neurological deficits were attributed to inadequate decompression, dural buckling, and spinal subdural hematoma. All patients regained normal neurological function after revision surgery in the current study.

#### 3.3.2 Short Term (6 Weeks–2 Years)

All five revision surgeries during this period occurred in adult patients, with causes including pseudarthrosis (one case) and implant failure (four cases).

#### 3.3.3 Intermediate Term (2–5 Years)

Among the 12 patients requiring revision during this period, the most common reasons were pseudarthrosis in six cases (four adults, two pediatric), implant failure in three cases (one adult, two pediatric), deformity progression in two pediatric cases, and infection in one adult patient.

#### 3.3.4 Long Term (> 5 Years)

Among the 11 patients requiring revision during this period, the most common reasons were deformity progression in seven cases (two adults, five pediatric), implant failure in three cases (one pediatric, two adults), and pseudarthrosis in one adult case.

**FIGURE 3:**
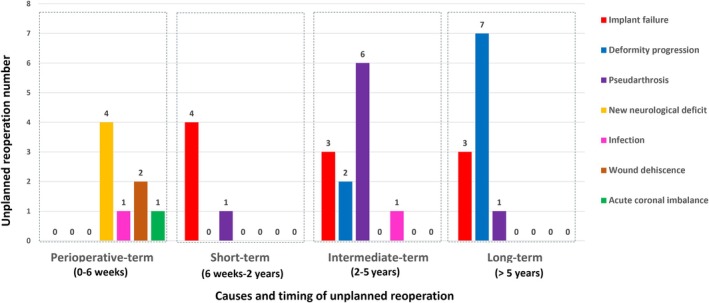
Distribution of unplanned reoperation following three‐column osteotomy in different time periods.

Additionally, seven of the eight patients (87.5%) with pseudarthrosis required reoperation for more than 2 years postoperatively. The detailed distribution of the causes and timing of unplanned revision surgeries in pediatric and adult patients is shown in Figure 3 and Table 2. Moreover, the detailed surgical revision strategies are presented in Table 3.

**TABLE 2:** Distribution of unplanned revision in pediatric and adult spinal deformity patients (*n* = 36).

Causes		Perioperative (0–6 weeks)	Short term (6 weeks–2 years)	Intermediate term (2–5 years)	Long term (> 5 years)	Overall
Pseudarthrosis	Overall	0	1	6	1	8
	Pediatric	0	0	2	0	2
	Adult	0	1	4	1	6
Deformity progression	Overall	0	0	2	7	9
	Pediatric	0	0	2	5	7
	Adult	0	0	0	2	2
Implant failure	Overall	0	4	3	3	10
	Pediatric	0	0	2	1	3
	Adult	0	4	1	2	7
New neurologic deficit	Overall	4	0	0	0	4
	Pediatric	2	0	0	0	2
	Adult	2	0	0	0	2
Wound dehiscence	Overall	2	0	0	0	2
	Pediatric	1	0	0	0	1
	Adult	1	0	0	0	1
Infection	Overall	1	0	1	0	2
	Pediatric	0	0	0	0	0
	Adult	1	0	1	0	2
Acute coronal imbalance	Overall	1	0	0	0	1
	Pediatric	0	0	0	0	0
	Adult	1	0	0	0	1
Overall	Overall	8	5	12	11	36
	Pediatric	3	0	6	6	15
	Adult	5	5	6	5	21

**TABLE 3:** Patients who underwent unplanned revision: Reason, time and surgical revision strategy.

**No**.	Age	Gender	Diagnosis	3CO level(s)	Fusion levels	Reasons for revision	Time to revision	Revision surgical strategy
1	31	Female	Congenital	VCR at L3	T11 to S1	Left S1 screw broken	7 months	Replace the broken screws and L5/S1 fusion
2	31	Male	Congenital	VCR at T11	T6 to L4	Right rod separated from the L2–L4 pedicle screws	1 year	Replace new connectors
3	44	Male	Congenital	VCR at T7	T2 to L2	Right T12 screw displacement	7 months	Remove the T12 screw
4	52	Male	Neuromuscular	PSO at T10	T4 to L4	Right L3 and L4 screws loosening	1 year and 5 months	Replace the screws
5	14	Male	Syringomyelia‐associated	VCR at T9	T3 to L3	Left T5 and T7 painful screws	2 years and 6 months	Remove the T5 and T7 screws
6	48	Female	Idiopathic	PSO at L1	T6 to L4	Right L4 screw broken and right rod broken	3 years and 1 months	Replace the broken rod; add additional rod
7	3	Female	Congenital	Hemivertebra resection at L3	L2 to L5	Right L5 painful screw	3 years and 4 months	Remove the right L5 screw
8	26	Male	Syringomyelia‐associated	SRS‐Schwab Grade 4 osteotomy at T9	T3 to L3	Bilateral rod broken	6 years and 3 months	Remove the part of broken rod
9	28	Female	Idiopathic	PSO at L2	T5 to L5	Right L4 and L5 screws broken	7 years and 2 months	Replace the broken screws
10	3	Male	Congenital	Hemivertebra resection at T12	T10 to L2	Left L2 screw broken	9 years and 10 months	Replace the broken screw
11	10	Female	Congenital	Hemivertebra resection at T5 and T6	T2 to T11	Deformity progression	2 years and 4 months	Posterior correction and extension of distal fusion to L4
12	8	Female	Congenital	Hemivertebra resection at T7	T5 to T9	Deformity progression	4 years and 4 months	Posterior correction and extension of fusion from T2 to L3
13	7	Female	Congenital	Hemivertebra resection at T10	T9 to T11	Deformity progression	6 years and 10 months	Posterior correction and extension of fusion from T8 to L4
14	25	Female	Congenital	PSO at T11	T3 to L4	Deformity progression	7 years and 2 months	Posterior correction with PSO at L2
15	5	Female	Congenital	Hemivertebra resection at T11 and L1	T10 to T12, L2 to L4	Deformity progression	7 years and 4 months	Posterior correction and extension of fusion from T7 to L4
16	6	Female	Congenital	Hemivertebra resection at T8	T6 to T10	Deformity progression	9 years and 8 months	Posterior correction and extension of fusion from T2 to L3
17	32	Male	Ankylosing spondylitis	VCR at L2	T11 to L5	Deformity progression	10 years and 1 months	Posterior correction and extension of fusion from T8 to S1; PSO at L4
18	7	Male	Congenital	Hemivertebra resection at T8 and T10	T7 to T11	Deformity progression	10 years and 8 months	Posterior correction and extension of fusion from T3 to L3
19	2	Male	Congenital	Hemivertebra resection at T7	T6 to T8	Deformity progression	10 years and 8 months	Dual growing rod techniques from T2 to L4
20	26	Female	Congenital	VCR at T12	T6 to L4	Pseudarthrosis (T10/T11)	1 year and 8 months	Posterior supplementary fusion; add additional rod
21	17	Male	Congenital	VCR at T11 and T12	T5 to L4	Pseudarthrosis (L1/2)	2 years and 6 months	Posterior supplementary fusion and extension from T4 to L4
22	21	Male	Idiopathic	PSO at T11	T2 to L4	Pseudarthrosis (L2/3)	2 years and 7 months	Posterior supplementary fusion
23	46	Female	Congenital	VCR at T9	T3 to L4	Pseudarthrosis (T10/11)	3 years and 2 months	Posterior supplementary fusion
24	39	Female	Idiopathic	PSO at T11	T4 to L5	Pseudarthrosis (L1/2, L2/3)	3 years and 3 months	Posterior supplementary fusion
25	11	Male	Congenital	PSO at T8	T4 to T11	Pseudarthrosis (T9/10)	3 years and 11 months	Posterior supplementary fusion and extension from T2 to L3
26	47	Male	Ankylosing spondylitis	PSO at T12 and L3	T8 to L5	Pseudarthrosis (L3/4)	4 years and 6 months	Posterior supplementary fusion and extension to S1
27	18	Female	Congenital	Hemivertebra resection at L5	T5 to S1	Pseudarthrosis (T12/L1, L1/2)	6 years and 3 months	Posterior supplementary interbody fusion at T12/L1 and L1/2
28	38	Female	Post‐tubercular spinal deformity	VCR at T10, T11 and T12	T5 to L4	New neurological deficit	2 h	Spinal subdural hematoma clearance
29	37	Male	Congenital	VCR at T12	T5 to L5	New neurological deficit	4 h	Decompression
30	12	Female	Congenital	VCR at T12	T1 to L3	New neurological deficit	4 h	Decompression
31	6	Male	Congenital	Hemivertebra resection at T5 and T6	T3 to T8	New neurological deficit	10 days	Decompression
32	25	Female	Congenital	PSO at T8	T2 to L4	Early infection	40 days	Irrigation and debridement, antibiotic therapy; without removal of implant
33	24	Female	Congenital	VCR at T11	T6 to L3	Later infection	2 years and 5 months	Irrigation and debridement, antibiotic therapy; without removal of implant
34	2	Male	Congenital	Hemivertebra resection at T12	T11 to L1	Wound dehiscence	11 days	Surgical wound re‐suturing
35	58	Female	Idiopathic	PSO at T9	T4 to L2	Wound dehiscence	12 days	Surgical wound re‐suturing
36	21	Female	Post‐tubercular spinal deformity	VCR at T8 and T9	T2 to L3	Acute coronal imbalance	33 days	Extension of distal fusion to pelvis

*Note: p* < 0.05 indicates statistical significance. Bold indicates statistically significant *p* values.

Abbreviations: PSO, pedicle subtraction osteotomy; VCR, vertebral column resection.

Overall, the unplanned reoperation rate was similar between pediatric (17.9%) and adult (15.9%) patients. However, the causes of reoperation differed: deformity progression was more common in pediatric patients, while implant failure and pseudarthrosis were more frequent in adults. Additionally, the timing of revisions also varied: pediatric revisions were more common in the intermediate and long‐term, whereas adult revisions were more evenly distributed across all time periods.

### 3.4 Risk Factors Analysis for Unplanned Revision

In this cohort, patients undergoing multilevel 3CO had significantly higher unplanned revision rates than those undergoing single‐level 3CO (33.3% vs. 14.6%, respectively; *p* = 0.02). However, no significant differences were observed between patients with and without unplanned revision surgery in terms of age, sex, operation time, number of fusion levels, estimated blood loss, or history of prior spinal fusion (*p* > 0.05) (Table 1).

## 4 Discussion

Our study addresses a critical gap in clinical literature by systematically elucidating the long‐term outcomes of unplanned reoperations after 3CO for spinal deformities—an important clinical issue that remains underexplored. The novelty of our extended follow‐up data provides a roadmap for time‐sensitive preventive strategies.

### 4.1 Implant Failure: The Leading Cause of Reoperation

We found that implant failure emerged as the most common cause of reoperation after 3CO, with an overall rate of 4.6%. Notably, no implant‐related revisions occurred during the perioperative period. Previous studies consistently identified implant failure as a significant issue following 3CO therapy. Smith et al. [22] reported rod breakage as one of the most common indications for revision in a multicenter study after 3CO. Similarly, Chan et al. [4] found that implant migration and broken implants were the primary reasons for revision in pediatric and adult deformity patients treated with 3CO. Maier et al. [13] documented instrumentation failure rates of 3% at 3 months and 4.5% at 1 year after 3CO surgery. More recently, Corbett et al. [11] reported that instrumentation failure accounted for 27.1% (13 of 48 patients) of revision surgeries after 3CO. Consistent with these findings, our study revealed a revision rate of 27.8% (10 of 36 patients) due to implant failure over the long‐term follow‐up period. These findings highlight the urgency of mitigating implant failure. Strategies such as satellite rod reinforcement may reduce mechanical complications [12, 17, 33, 35, 44, 45, 46, 47, 48, 49, 50, 51].

### 4.2 Deformity Progression: A Predominant Issue in Pediatric Patients

In this paper, deformity progression was another leading cause of unplanned reoperations, with an incidence rate of 4.2%. Notably, 77.8% of these cases occurred in pediatric spinal deformity patients, particularly in those with congenital spinal deformities. This finding aligns with those of previous studies highlighting deformity progression as a significant reason for revision surgery after 3CO. Chan et al. [4] reported that two of 32 congenital spinal deformities required reoperation following 3CO due to scoliosis progression. Similarly, Wang et al. [16] documented one case of progressive deformity requiring unanticipated surgery among 36 congenital scoliosis patients after posterior hemivertebral resection. Chang et al. [52] found that 3 of 45 pediatric congenital scoliosis (6.7%) required revision after posterior VCR. Importantly, our study revealed that deformity progression was the most common reason for long‐term unplanned revision surgery after 3CO surgery. These findings underscore the necessity for close postoperative follow‐up and thorough preoperative discussions with patients and their families regarding the risk of revision surgery due to deformity progression, especially in pediatric patients with congenital spinal deformities [53, 54].

### 4.3 Pseudarthrosis: A Delayed Complication More Common in Adults

Our results also identified pseudarthrosis as one of the most common causes of unplanned reoperations, with a rate of 3.7%. We found that 75% of pseudarthrosis cases occurred in adult spinal deformities. Additionally, 87.5% of pseudarthrosis cases were diagnosed more than 2 years after the 3CO procedures. Indeed, several prior studies have described unplanned reoperation due to pseudarthrosis following 3CO [11, 13, 55, 56]. Kim et al. [25] reported that half of 10 pseudarthrosis cases were identified within 2 years after PSO for fixed sagittal imbalance, whereas the remaining cases were detected between 2 and 4 years postoperatively. Similarly, Chan et al. [4] documented two cases of pseudarthrosis requiring reoperation among 32 patients with spinal deformity treated with 3CO. O'neill et al. [5] further reported that 42% of pseudarthrosis cases were identified more than 2 years postoperatively. Collectively, these findings emphasize the importance of long‐term follow‐up after 3CO and underscore the need for surgeons to remain vigilant in monitoring and addressing pseudarthrosis, particularly in adults. Strategies to reduce or prevent pseudarthrosis‐related revisions should be prioritized, as supported by previous research [12, 25, 30, 56, 57].

### 4.4 Neurological Deficits: An Early and Critical Concern

Our data revealed that the incidence of unplanned revisions due to new neurological deficits was 1.9%. It is important to note that new neurological deficits were the most common reason for revision during the perioperative period. Several previous studies have consistently highlighted the risk of neurological deficits following 3CO surgery [19, 26, 27, 29], including complete permanent neurological deficit [20, 21]. In the prospective Scoli‐Risk‐1 analysis, Kelly et al. [19] reported a 9.9% rate of new neurological deficits after 3CO. Similarly, Riley et al. [6] documented a 9.3% rate of neurological deficits in 54 pediatric and adult spinal deformity patients following posterior VCR. Maier et al. [13] reported revision rates due to neurological deficits of 1.8% at 3 months and 2.1% at 1 year postoperatively in 335 adult patients with spinal deformity. More recently, in 2024, Lafage et al. [58] found that neurological deficits typically develop early postoperatively, with the highest incidence occurring within the first 60 days. Consistent with these findings, our study demonstrated that all revisions due to new neurological deficits (four cases) occurred during the perioperative period, including three cases requiring immediate revision on the same day as the 3CO procedure and one case requiring reoperation 10 days postoperatively. Importantly, all patients achieved normal neurological function during follow‐up after revision surgery. These results further emphasize the critical importance of closely monitoring neurological function in the perioperative period (0–6 weeks), particularly on the same day as 3CO surgery, as emphasized in previous research [33].

### 4.5 Other Reasons: Wound Dehiscence, Infection, and Coronal Imbalance

We found that additional reasons for unplanned revision included wound dehiscence (0.93%), infection (0.93%), and acute coronal imbalance (0.46%). These findings agree with those of previous studies that have similarly identified wound dehiscence, infection, and acute coronal imbalance as notable causes of unplanned reoperation after 3CO [5, 6, 11, 13, 59]. Collectively, our results highlight that these complications, though less frequent, should not be overlooked in the postoperative management of 3CO patients.

### 4.6 Multilevel 3CO: Special Attention Should Be Paid

Our analysis revealed that patients who underwent multilevel 3CO had a significantly higher unplanned revision rate than those who underwent single‐level 3CO. Multilevel 3CO may exacerbate load‐shifting effects across osteotomy sites, increasing mechanical strain on implants and adjacent segments [45]. In this paper, no significant associations were found between unplanned revision and factors such as age, sex, operative time, number of fusion levels, estimated blood loss, or a history of prior spinal fusion. These findings are consistent with those reported by Maier et al. [13] in their multicenter study. Therefore, our data suggest that special attention should be paid when multilevel 3CO is performed.

### 4.7 Strengths and Limitations

The strengths of this study include its long‐term follow‐up (minimum 8 years), which provides a comprehensive temporal analysis of reoperations beyond the 5‐year horizon that is rarely captured in existing studies. Furthermore, the inclusion of both pediatric and adult populations with diverse etiologies from a high‐volume single center enhances the generalizability of our findings across a broad spectrum of spinal deformity patients. The application of a standardized classification system for the timing of reoperations allows for meaningful comparisons with future studies [6]. We believe these findings enhance our understanding of unplanned revisions following 3CO and offer valuable insights to guide surgical decision‐making, revision strategy, improve patient counseling, and support efforts by clinicians managing spinal deformities in both pediatric and adult populations to optimize the cost‐effectiveness of 3CO procedures.

However, several limitations should be acknowledged. First, the retrospective design and lack of prospective data collection may have introduced potential bias. Second, because all patients were recruited from a single medical center, the generalizability of our findings may be limited. Third, we did not investigate the impact of unplanned revision surgery on health‐related quality of life outcomes [34, 59, 60, 61, 62, 63]. Fourth, due to the retrospective nature of the study, important potential confounding variables such as bone mineral density [57, 64], detailed radiographic parameters, such as spinopelvic alignment [13], and specific comorbidities, such as a history of deep vein thrombosis [31], chronic steroid use [24], were not systematically collected or analyzed. Fifth, the sole parameter of significance was the “number of 3CO level,” with no other variables demonstrating meaningful associations in our current study. Consequently, multivariate regression analysis was not performed. Future prospective, multicenter studies are warranted to validate our findings, incorporate patient‐reported outcomes, and control for a broader range of potential confounders through advanced statistical modeling. Such studies should prioritize the systematic collection of bone mineral density, detailed radiographic measures, and comorbidity data to further elucidate the multifactorial nature of unplanned reoperations after 3CO.

## 5 Conclusion

In this study, the overall unplanned reoperation rate following 3CO for pediatric and adult spinal deformities was 16.7%. Implant failure, deformity progression, and pseudarthrosis were the most common reasons. Future research could particularly investigate ways to prevent the top causes, like satellite rods for multilevel 3CO, improved fusion techniques, or closer monitoring for deformity progression. Overall, we provide useful data for surgeons performing 3CO, highlighting the importance of long‐term surveillance and proactive strategies to mitigate revision risks, particularly in patients with multilevel 3CO.

## Author Contributions

**Youping Tao:** conceptualization; data curation; investigation; methodology; writing – original draft; writing – review and editing. **Chenhao Zhao, Jiaxu Wang, Bo Gao, Haixia, Li, Yongyu Hao, Litao Huo, Shibo Huang, Zhiming Chen, Shuwei Ma, Shuilin Shao:** investigation; methodology; writing – review and editing. **Jigong Wu:** conceptualization; investigation; methodology; supervision; writing – review and editing and take responsibility for the integrity of the work as a whole from inception to published article.

## Ethics Statement

This study was approved by our institutional ethics review committee (ethical approval number: LL‐LCSY‐2024‐14) and complied with the Declaration of Helsinki.

## Conflicts of Interest

The authors declare no conflicts of interest.

## Data Availability

The data that support the findings of this study are available from the corresponding author upon reasonable request.
